# Tooth Bleaching: A bibliometric analysis of the top 100 most-cited papers

**DOI:** 10.1590/0103-6440202305290

**Published:** 2023-05-15

**Authors:** Aurélio de Oliveira Rocha, Lucas Menezes dos Anjos, Filipe Colombo Vitali, Pablo Silveira Santos, Michele Bolan, Carla Miranda Santana, Mariane Cardoso

**Affiliations:** 1 Department of Dentistry, Federal University of Santa Catarina, Florianopolis (Santa Catarina), Brazil.

**Keywords:** tooth whitening, tooth bleaching, hydrogen peroxide, carbamide peroxide, bibliometric analysis

## Abstract

This study analyzes the characteristics of the top 100 most-cited papers related to tooth bleaching. A literature search was performed on the Web of Science up to March 2022. The number of citations was cross-matched with the citation count on Scopus and Google Scholar. The following data were collected: number and density of citations; authorship; year and journal of publication; study design and thematic; keywords; institution and country of origin. Spearman’s correlation and Poisson regression were used to determine associations between the number of citations and study characteristics. The VOSviewer software was used to generate collaborative network maps for the authors and keywords. The number of citations ranged from 66 to 450. Papers were published between 1981 and 2020. The most frequent study design and topic were laboratory-based studies and ‘interaction of the bleaching agent with dental tissues’, respectively. Cochran M, Loguercio AD, Matis B, Reis A, and Suliman M were the authors with the highest number of papers. The countries with the most papers were the United States of America (USA) (28%) and Brazil (20%). Indiana University and State University of Ponta Grossa were the institutions with the most papers (6% each). There was a very strong correlation among the number of citations of the three databases. The 100 most-cited papers related to tooth bleaching were mainly published by the USA and Brazil, with laboratory-based studies addressing topics related to the effects of bleaching agents on tooth structure being the most prevalent.

## Resumo

Este estudo analisa as características dos 100 artigos mais citados relacionados ao clareamento dental. Uma pesquisa bibliográfica foi realizada na Web of Science até março de 2022. O número de citações foi cruzado com a contagem de citações na Scopus e na Google Scholar. Foram coletados os seguintes dados: número e densidade de citações; autoria; ano e periódico de publicação; desenho do estudo e temática; palavras-chave; instituição e país de origem. A correlação de Spearman e a regressão de Poisson foram usadas para determinar associações entre o número de citações e as características do estudo. O software VOSviewer foi utilizado para gerar mapas de rede colaborativa para os autores e palavras-chave. O número de citações variou de 66 a 450. Os artigos foram publicados entre 1981 e 2020. O desenho e o tópico de estudo mais frequentes foram estudos laboratoriais e interação do agente clareador com tecidos dentários, respectivamente. Cochran M, Loguercio AD, Matis B, Reis A e Suliman M foram os autores com maior número de artigos. Os países com mais artigos foram os Estados Unidos da América (EUA) (28%) e o Brasil (20%). A Universidade de Indiana e a Universidade Estadual de Ponta Grossa foram as instituições com mais artigos (6% cada). Houve uma correlação muito forte entre o número de citações das três bases de dados. Os 100 artigos mais citados relacionados ao clareamento dentário foram publicados principalmente pelos EUA e Brasil, sendo os estudos laboratoriais que abordaram tópicos relacionados aos efeitos dos agentes clareadores na estrutura dentária os mais prevalentes.

## Introduction

In recent years, there has been a growing consumption of aesthetic dental procedures to obtain a harmonic smile with white and aligned teeth ^1^ ^,^ ^2^. In this sense, tooth bleaching has experienced an increase in clinical practice, as it is a conservative, safe, effective, and less expensive aesthetic procedure that offers satisfactory results in the short term ^1^. A previous study carried out in Brazil reported that the prevalence of tooth bleaching at 31 years was 15.6%, while 85.9% of the interviewed patients reported desiring the treatment ^2^.

Tooth bleaching is a process of discoloration or whitening that occurs on the tooth surface as a result of the action of a bleaching agent ^3^. In general, bleaching agents act as oxidants that diffuse into the tooth surface and dissociate to produce free radicals, which will act by destroying one or more bonds in the conjugated chain of colored organic molecules that darken the tooth (chromophore) ^1^ ^,^ ^4^. This process results in smaller and less pigmented molecules, leading to tooth bleaching. The field of research on tooth bleaching is classic in the scientific literature. The first known report on tooth bleaching was published by Chapple in 1877 using oxalic acid ^5^. Despite a large number of published studies on this topic, tooth bleaching is still a frequent topic of study, mainly related to the efficacy of the protocols, the action of bleaching agents, the longevity of the results, and the possibility of dental damage ^3^ ^,^ ^5^.

Bibliometric analysis is a research method that uses specific tools, including mathematical methods, to quantitatively analyze the main characteristics of papers published in a specific area of ​​knowledge ^6^. In this way, it is possible to assess the impact and growth of scientific production and tendencies in a specific field. The most used method in the bibliometric analysis consists of analyzing the number of citations of a paper over time ^6^ ^,^ ^7^. The topics and designs of highly cited papers have the potential to influence tendencies in clinical practice and future research ^7^.

Specifically in dentistry, previous studies that conducted bibliometric analyses allowed us to understand the scientific status of different topics relevant to clinical practice, such as dental caries, erosive tooth wear, and laser (7-9). However, to the best of our knowledge, no previous bibliometric review has evaluated the main characteristics of papers that address tooth bleaching as a topic of study. Due to the frequent worldwide search for aesthetic purposes, this topic must be scientifically investigated to ensure greater safety, technical improvement, and clinical access. Therefore, this paper aimed to identify and analyze the main characteristics of the top 100 most-cited papers related to tooth bleaching, helping researchers to identify trends for future research in this field of study. 

## Methodology

An electronic search was conducted in March 2022 in the Web of Science Core Collection (WoS-CC) database (https://www.webofscience.com). To select the papers, the following search strategy was applied: [TS= (“dental whitening” OR “tooth whitening” OR “teeth whitening” OR “tooth bleaching” OR “teeth bleaching” OR “dental bleaching” OR “tooth bleaching agents” OR “bleaching materials” OR (bleaching AND teeth))]. No filters, date, or language restrictions were applied. Conference papers and studies in which tooth bleaching was not linked to the main theme were excluded. 

The identified papers were organized in descending order considering the absolute number of citations. The top 100 most-cited papers were selected by two independent reviewers after reading the title, abstract and full text, when necessary. Disagreements were resolved by consensus with a third reviewer. Subsequently, the number of citations of each selected paper was cross-checked with the number of citations in the Scopus (https://www.scopus.com) and Google Scholar (https://scholar.google.com.br) databases. In case of a tie in the number of citations, the position of a paper in the list was based on the highest density (number of citations per year) of WoS-CC citations. 

The following bibliometric data were extracted from each paper: title; contributing authors; year and journal of publication; number and density of citations; institution, country, and the continent of origin (based on the affiliation of the corresponding author); impact factor of the journal in 2021 (according to the Journal Citation Reports); keywords; study design and topic of study. The study design was classified as follows: systematic reviews, literature reviews, case reports, laboratory-based, observational, and interventional studies ^7^. Considering the topic of study, the papers were grouped according to the most prevalent topics, as follows: techniques and agents for bleaching, interaction with dental tissues (diffusion and compromise), bleaching with light, adverse effects (tooth sensitivity, resorption, or toxicity), and the relationship between the bleaching agent and restorative materials. Topics that were addressed in a single study were classified as “others”.

To identify and determine the number of occurrences/citations of keywords and contributing authors, a specific command of the Visualization of Similarities Viewer (VOSviewer, version 1.6.17.0, Netherlands) was used. The other data were manually identified and counted. All data were double-checked by two independent reviewers. Additionally, VOSviewer was used to generate a graphical representation of existing bibliometric networks, identifying the connections among authors (only connected authors were presented), as well as the most prevalent keywords (only keywords with at least three occurrences were included). In the networking analysis, the terms corresponding to the largest foci and fonts, and more evident colors (close to red) showed greater occurrence or correlation between the terms. On the other hand, the lower the focus, font, or color tone (closer to yellow), the lower the occurrence of the associated term.

*Spearman*’s correlation was applied to determine if there were correlations between the number of citations among the searched databases. Prior to analysis, the *Kolmogorov-Smirnov* test was used to verify the normality of the data distribution - due to non-normal distribution, *Spearman's* correlation coefficient test was used. In addition, *Poisson* regression was performed to determine associations between the total number of WoS-CC citations with ‘study design’, ‘continent’, ‘year of publication’, and ‘journals impact factor’. Data analysis was performed using the statistical software SPSS for Windows (SPSS, version 24.0; IBM Corp).

## Results

### Search results

The search strategy resulted in a total of 2,269 papers that were organized in descending order of the number of citations. To identify the top 100 most-cited papers related to tooth bleaching, the first 119 documents were analyzed, of which 19 were excluded for different reasons: two were conference papers and 17 did not address the proposed topic. After the selection process, the top 100 most-cited papers related to tooth bleaching were identified (Box 1).

**Box 1:**
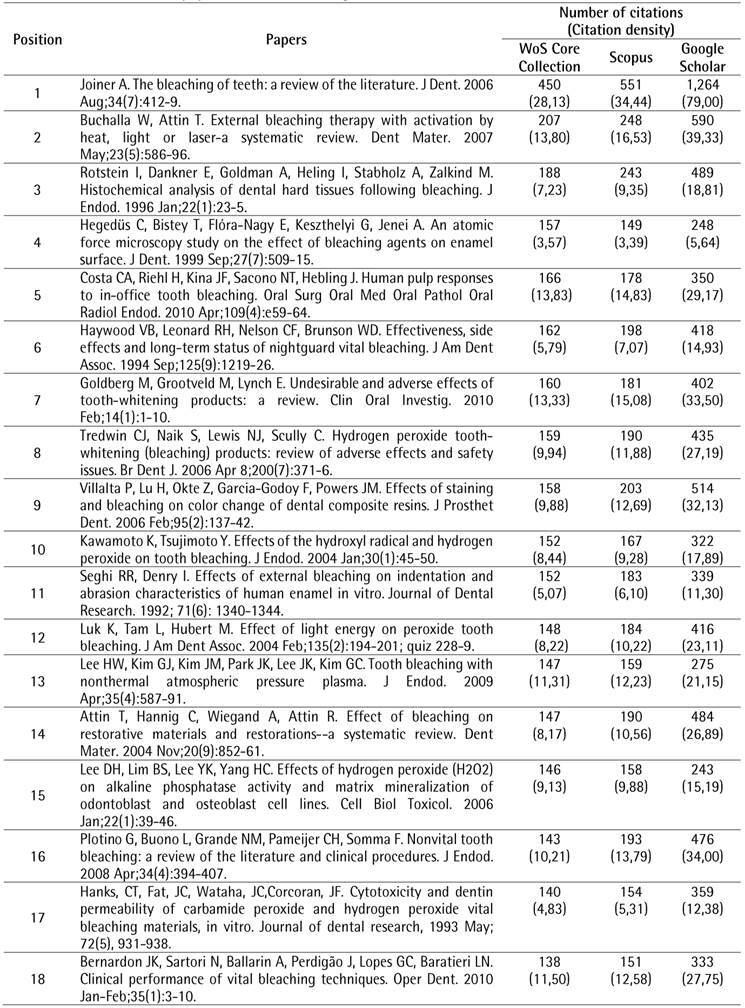
The 100 most-cited papers in tooth bleaching.

**Box 1:**
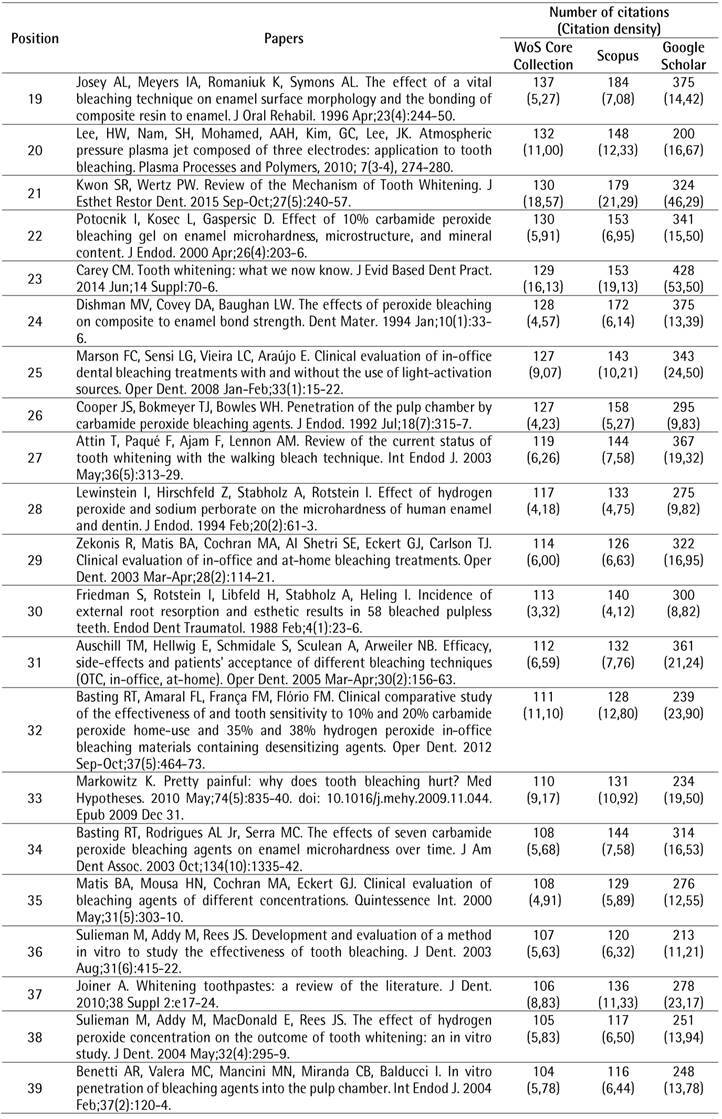
Continuation

**Box 1:**
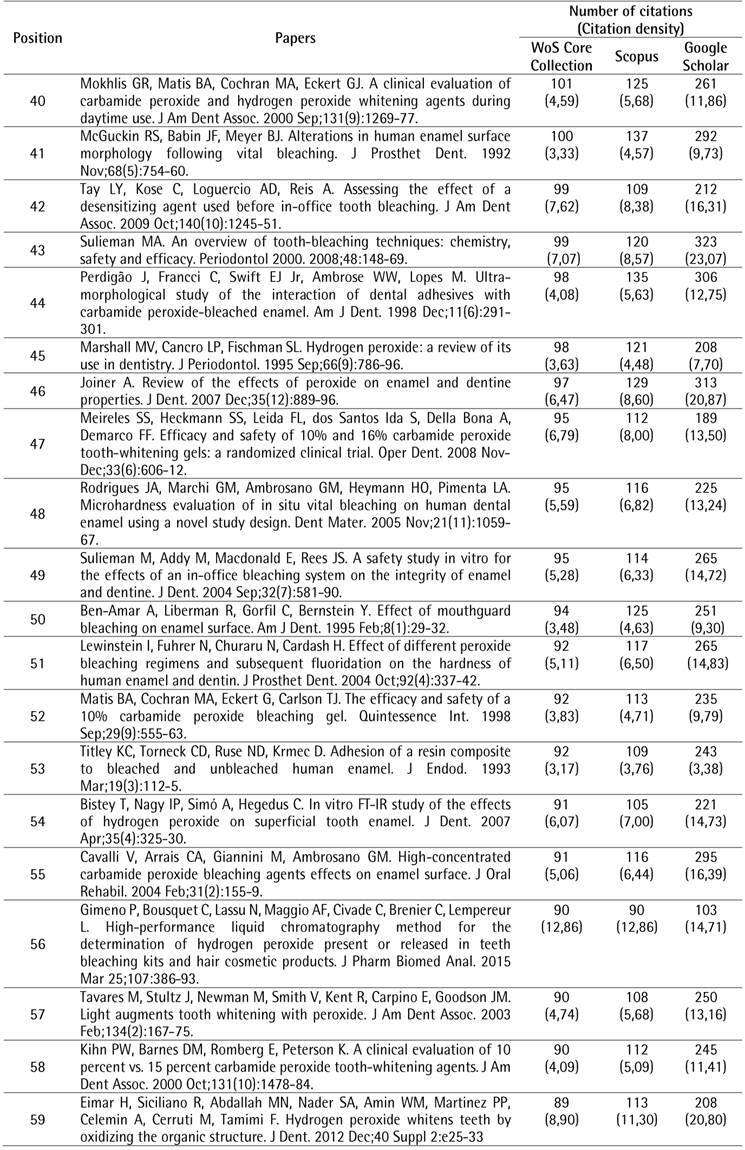
Continuation

**Box 1:**
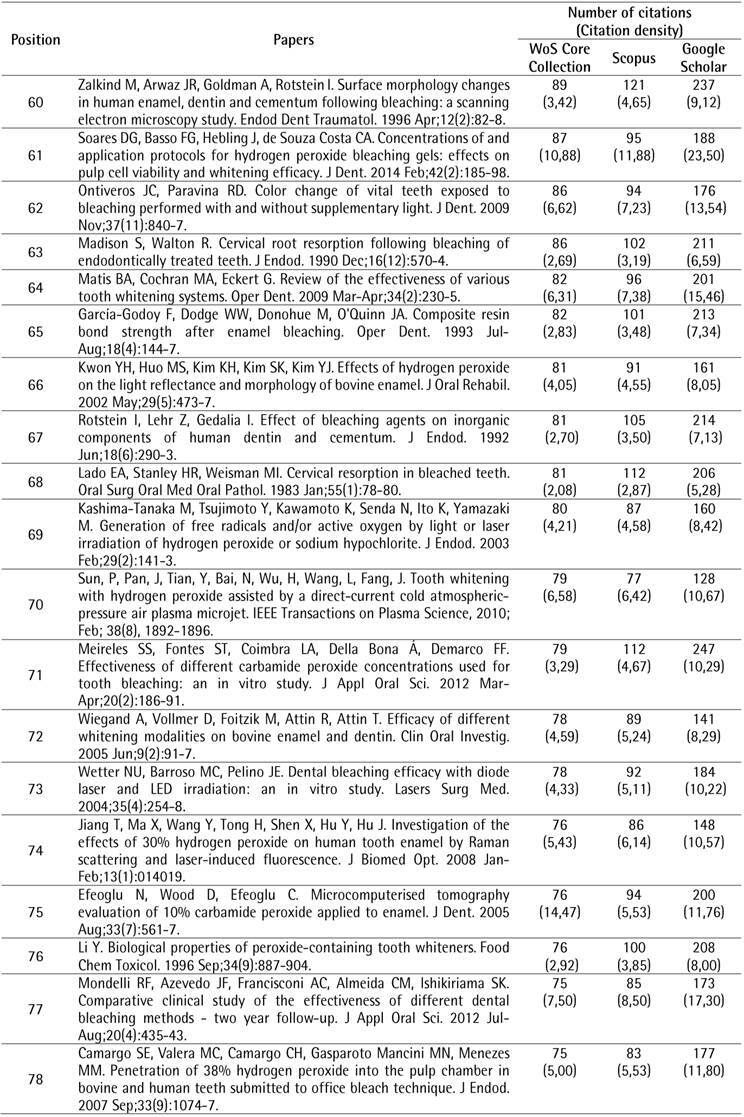
Continuation

**Box 1:**
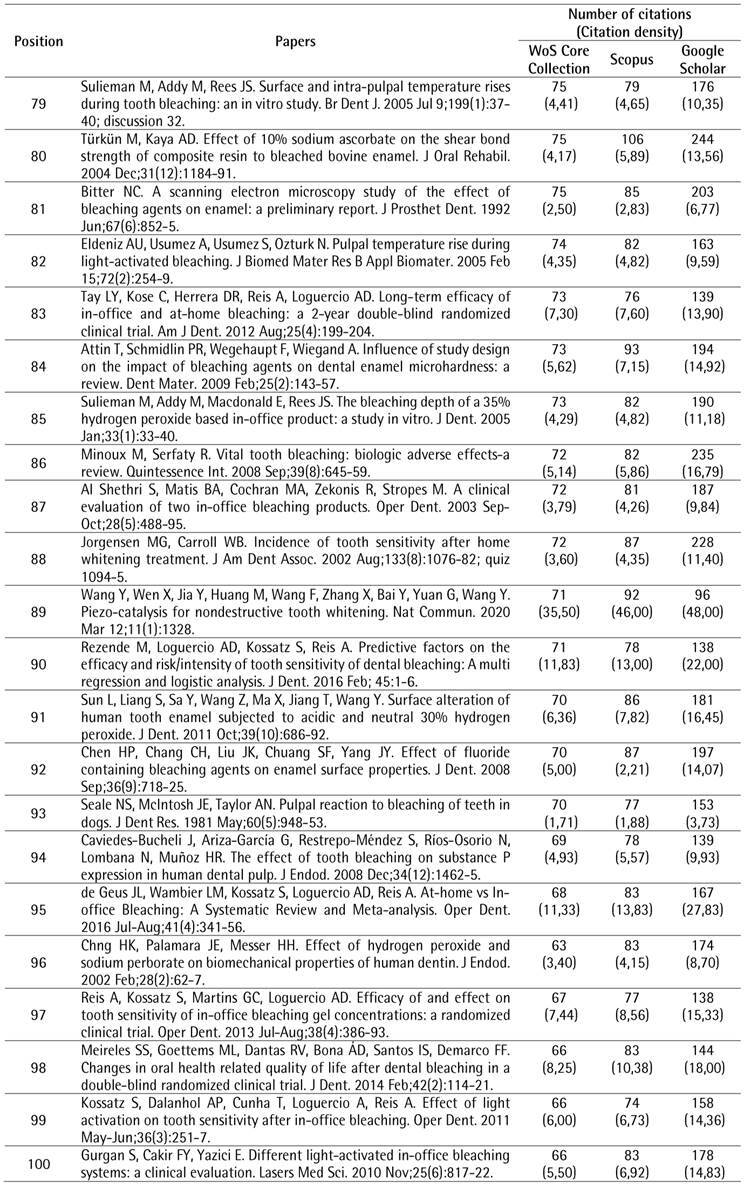
Continuation

### Citation analysis

The top 100 papers received a total of 10,591 citations in the WoS-CC. The number of citations ranged from 66 to 450. Self-citations accounted for 5.54% (n = 587) of WoS-CC citations. Forty-one papers were cited more than 100 times. In the other two databases, a greater number of citations were observed, with 12,612 (ranging from 74 to 551) in Scopus and 25,578 (ranging from 96 to 1,264) in Google Scholar. There was a strong positive correlation between the number of citations in the WoS-CC and Google Scholar (rho = 0.868) and a very strong correlation in the WoS-CC and Scopus (rho = 0.960). 

The most-cited paper ^(^ ^4^ in the WoS-CC was “The bleaching of teeth: A review of the literature”, a literature review, published in the *Journal of Dentistry*, accumulating a mean of 28.13 citations per year, being also the most-cited in Scopus (551 citations) and Google Scholar (1,264 citations). The second most-cited paper ^(^ ^10^ in the WoS-CC was titled "External bleaching therapy with activation by heat, light or laser-a systematic review", a systematic review, published in *Dental Materials*, accumulated a mean of 13.80 citations per year, being also the second most cited in Scopus (248 citations) and Google Scholar (590 citations). The third most-cited paper ^11^ in the WoS-CC was "Histochemical analysis of dental hard tissues following bleaching", a laboratory study, published in the *Journal of Endodontics*, which accumulated an average of 7.23 citations per year, being also the third most cited in Scopus (243 citations) and Google Scholar (489 citations).

The paper with the highest density of citations in WoS-CC (35.50) was "Piezo-catalysis for nondestructive tooth whitening" ^(^ ^12^, published in the journal *Nature Communications*. The second paper with the highest density of citations in the WoS-CC (28.13) was "The bleaching of teeth" ^(^ ^4^, published in the *Journal of Dentistry*. The third paper with the highest density of citations in WoS-CC (18.57) was "Review of the Mechanism of Tooth Whitening" ^(^ ^13^, published in the *Journal of Esthetic and Restorative Dentistry*.

### Year of publication

**Figure 1:**
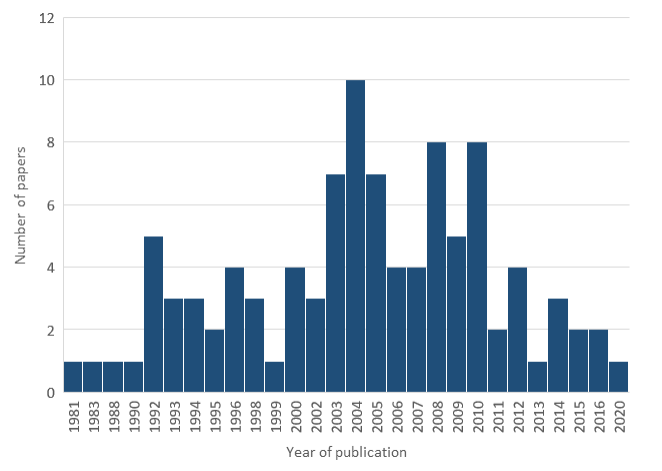
Distribution of the number of publications among the years.

 The oldest paper ^(^ ^14^ was published in 1981, titled “Pulpal reaction to bleaching of teeth in dogs”, and authored by Seale and collaborators. The most recent paper ^(^ ^12^ was published in 2020, titled “Piezo-catalysis for nondestructive tooth whitening”, and authored by Wang and collaborators. The largest number of the 100 most-cited papers (56%) was published in the 2000s, followed by the decades of the 1990s (25%), 2010s (15%), and 1980 (4%). The description of the number of publications each year can be seen in Figure 1. The *Poisson* regression analysis showed that the number of citations from WoS-CC tended to decrease by 0.7% each year (RR: 0.993, 95% CI: 0.990-0.996, p < 0.01) (Table 1).

**Table 1:** *Poisson* regression between the total number of WOS-CC citations and independent variables

Independent variables	WOS-CC number of citations	
	RR (95% CI)	*p-value*
Study design		
Laboratorial	1	
Interventional	1.253 (0.995 - 1.578)	0.055
Case report	1.314 (1.048 - 1.646)	0.018*
Literature review	1.551 (1.227 - 1.961)	<0.01*
Systematic review	1.628 (1.261 - 2.102)	<0.01*
Continent		
North America	1	
Europe	1.202 (1.130 - 1.279)	<0.01*
Asia	1.006 (0.945 - 1.072)	0.847
Oceania	0.877 (0.748 - 1.029)	0.107
South America	0.952 (0.891 - 1.018)	0.149
Year of publication	0.993 (0.990 - 0.996)	<0.01*
Journals’ impact factor	1.006 (0.996 - 1.016)	0.233

Legend: (CI) confidence interval, (RR) rate ratio, (WOS-CC) Web of Science Core Collection

### Contributing journals and impact factor

The journals in which the 100 most-cited papers were published are shown in Table 2. The *Journal of Dentistry* was the top one, with 17 papers (1,922 citations), followed by the *Journal of Endodontic*s (14%) (1,555 citations) and *Operative Dentistry* (12%) (1,134 citations). According to the journal citation reports, the journals with the highest impact factor (IF) in 2021 linked to dental clarification were linked: *Nature Communications* with one paper (IF 17,694; 71 citations), *Periodontology 2000* with one paper (IF 12,239; 99 citations) and *Journal of Dental Research* with three articles (IF 8,924; 362 citations). Poisson regression analysis showed no association between the number of citations and the journal’s impact factor (p > 0.05).

**Table 2:** Top 10 of the journals with the most papers in the 100 most-cited list.

Source Title	Number of papers	Number of citations	Impact factor
Journal of Dentistry	17	1,922	4,991
Journal of Endodontics	14	1,555	4,422
Operative Dentistry	12	1,134	2,937
The Journal of The American Dental Association	8	870	3,454
Dental Materials	5	650	5,687
Journal of Oral Rehabilitation	4	384	3,558
Quintessence International	4	351	2,175
Journal of Prosthetic Dentistry	4	425	4,148
American Journal of Dentistry	3	265	1,748
Journal of Dental Research	3	362	8,924

### Study design and topics

Most papers were laboratory-based (55 papers; 5,682 citations), followed by interventional studies (24 papers; 2,290 citations), literature reviews (17 papers; 2,116 citations), systematic reviews (3 papers; 421 citations), and case report (1 paper; 81 citations). The *Poisson* regression analysis showed that the number of citations was influenced by the study design (Table 1). Overall, case reports, literature reviews, and systematic reviews received more citations than laboratory-based studies. 

Most studies addressed the topic ‘interaction of bleaching agents with dental tissues’ (diffusion and complications) (38 papers; 3,879 citations), followed by ‘techniques and agents for bleaching’ (27 papers; 3,065 citations), ‘adverse effects’ (tooth sensitivity, resorptions, and toxicity) (14 papers; 1,417 citations), ‘use of light sources’ (9 papers; 948 citations), ‘the relationship between the bleaching agent and restorative materials’ (7 papers; 780 citations), and ‘other topics’ (5 papers; 502 citations). 

### Countries and continents

A total of 18 countries contributed to the top 100 most-cited papers. Considering the number of publications per country, the top five countries were: [1] the United States of America with 28 papers and 2,916 citations [2] Brazil with 20 papers and 1,860 citations; [3] the United Kingdom with 11 papers and 1,442 citations, [4] Israel with 7 papers and 774 citations, and [5] China with 5 papers and 366 citations.

**Figure 2:**
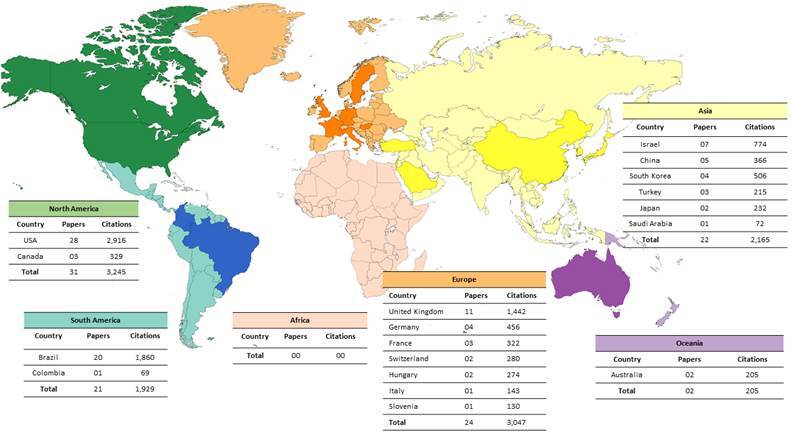
Map of countries and continents present among the top 100 most-cited papers.

Among the continents with the most papers in the top 100 (Figure 2), North America stands out (31 papers), which consequently has the highest number of citations in WoS-CC ^3^ ^,^245), Scopus ^3^ ^,^979) and Google Scholar ^8^ ^,^594); followed by Europe (24 papers; 3,047 citations), followed by Asia (22 papers; 2,165 citations), South America (21 papers; 1,929 citations) and Oceania (2 papers; 205 citations). The *Poisson* regression analysis showed that the number of citations of studies from Europe was 20.2% higher than the studies from North America (RR: 1.202, 95%CI: 1.130-1.279, p < 0.01) (Table 1).

### Contribution institutions

A total of 59 institutions contributed to the top 100 most-cited papers. Table 3 shows the top 10 institutions with the highest number of publications. The top three belong to Indiana University (United States of America) with 6 papers and 573 citations, the State University of Ponta Grossa (Brazil) with 6 papers and 444 citations, and The Hebrew University of Jerusalem (Israel) with 5 papers and 588 citations.

**Table 3:** Top 10 of the institutions with the highest number of papers among the 100 most-cited.

Institution	Country	Number of papers	Number of citations
Indiana University	USA	6	459
State University of Ponta Grossa	Brazil	6	444
The Hebrew University of Jerusalem	Israel	5	588
University of Bristol	United Kingdom	5	479
University State Paulista	Brazil	4	432
Unilever Oral Care	United Kingdom	3	812
University of Göttingen	Germany	3	344
University of North Carolina	USA	3	263
University of São Paulo	Brazil	3	261
University of Zurich	Switzerland	2	280

### Keywords

**Figure 3:**
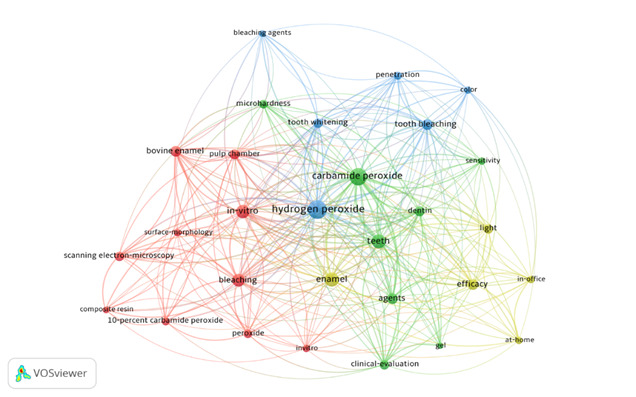
Density map of the most used keywords among the 100 most-cited papers.

A total of 341 keywords were identified in the top 100 most-cited papers. The most prevalent was ‘hydrogen peroxide’ (41%), followed by ‘carbamide peroxide’ (35%), ‘teeth’ (30%), and ‘in vitro’ (20%). Figure 3 shows the most prevalent keywords and the relationship between them. The keywords that correspond to the largest foci and that appear written with a highlighted font are the words that had the highest occurrence, on the other hand, the words that appear in the smallest foci had a lower occurrence. The keywords that are connected by the bibliometric networks are terms that showed a relationship between the studies.

### Contributing authors

**Figure 4:**
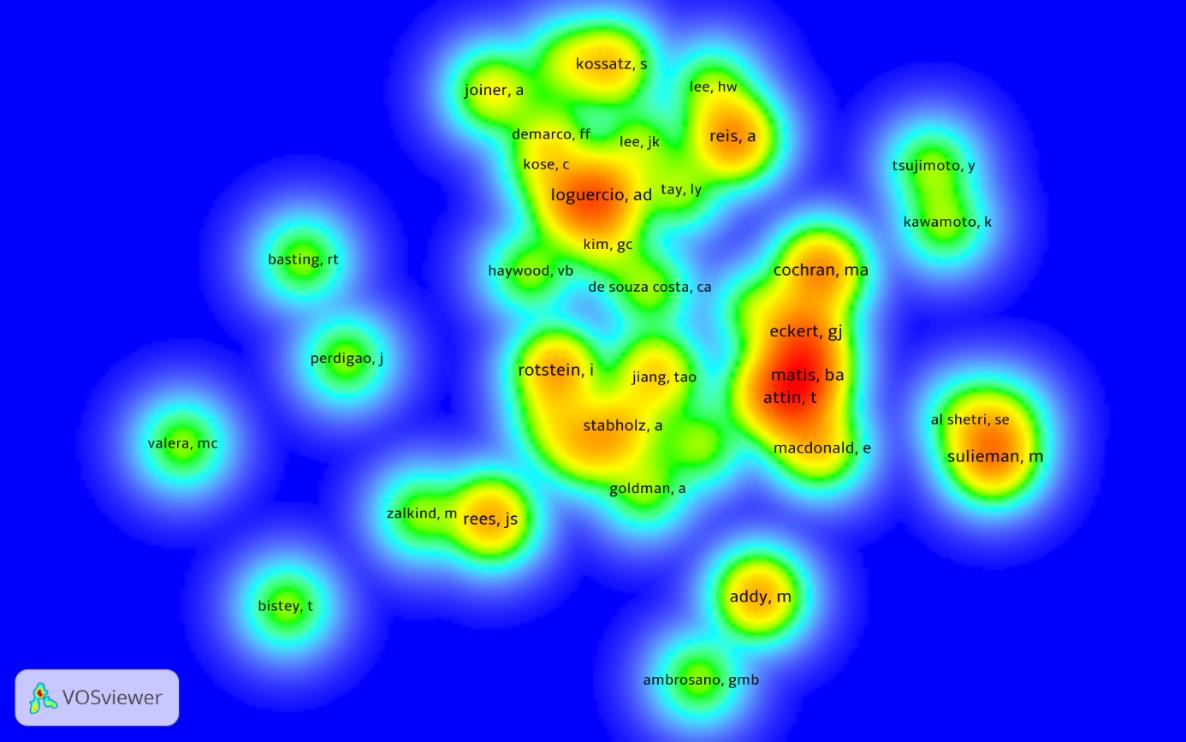
Density map of authors and collaborative co-authorship among them.

A total of 316 authors contributed to the top 100 most-cited papers. In Table 4, the top 10 authors with the highest number of publications are presented. Cochran MA, Matis BA, Sulieman M, Loguercio AD, and Reis A authored six documents each; Attin T, Rotstein I, Eckert GJ, Addy M, and Rees JS authored five documents each. The frequency they appear and the co-authorship relationship between them is represented in Figure 4. Names written with a highlighted font and corresponding to red/orange coloring are associated with the most frequent authors. On the other hand, the names associated with the green/blue colors correspond to the authors with a lower occurrence. 

**Table 4:** Top 10 authors with the most papers among the 100 most-cited.

Authors	Number of papers between 100 most-cited	Number of citations between 100 most-cited	Number of papers in WoS-CC	Number of citations in WoS-CC	H-Index
Cochran MA	6	569	72	2,299	28
Matis BA	6	569	72	1,562	23
Sulieman M	6	554	10	677	9
Loguercio AD	6	444	411	9,432	55
Reis A	6	444	217	3,530	29
Attin T	5	624	446	11,328	53
Rotstein I	5	588	101	2,535	28
Eckert GJ	5	497	494	8,581	47
Addy M	5	455	454	11,248	56
Rees JS	5	455	60	1,505	21

## Discussion

Since Alan Pritchard defined the concept of ‘bibliometrics’ in 1969 ^(^ ^15^, several bibliometric analyzes have been developed in the field of dentistry ^(^ ^7^ ^,^ ^8^ ^,^ ^9^ ^,^ ^16^. However, to the best of our knowledge, no bibliometric review analyzed the main characteristics of the top 100 most-cited papers related to tooth bleaching. Although this bibliometric analysis is a current study, the compilation of the 100 most-cited papers gathers research conducted over 40 years ago and which, due to a large number of citations, has become important for the topic. Thus, the 100 most-cited papers in the literature related to tooth bleaching, indexed in WoS-CC, were identified in this paper. It was observed that the scientific progress of this topic is supported mainly by laboratory-based studies, analyzing the interaction of bleaching agents and their diffusion and effects on the dental structure.

Papers that received more than 100 citations in a specific area can be considered a classic ^(^ ^17^. Thus, the present review highlights that 41 of the most-cited papers surpass this metric, demonstrating its importance and contribution to scientific development in this area. In addition, 5.54 were self-citations, a considerably high number when compared to other bibliometric reviews. However, although the practice of self-citation is not well interpreted, it can occur because of the great contribution of certain authors in a specific field of knowledge ^18^.

The most-cited paper was ‘The bleaching of teeth: A review of the literature’, authored by Joiner A in 2006 ^4^. The purpose of this study was to review the knowledge about external tooth bleaching and factors influencing the efficacy of the whitening process. From the 2000s, the growing demand for whiter teeth exponentially increased the clinical demand for tooth bleaching, as well as the demand for dental treatments ^(^ ^19^. In the same trend, tooth whitening products are popularly marketed as oral care products ^(^ ^19^. Thus, the years between 2001 and 2010 were the period that concentrated the largest number of papers in this bibliometric analysis. Due to the various questions about techniques and the emergence of new materials for tooth bleaching, investigating the current situation of this theme becomes fundamental ^(^ ^19^ ^,^ ^20^. In addition to this study, Joiner A has engaged in other publications on this topic, also appearing as the first author in two other papers of this top 100 ^21^.

Regarding the contribution countries, the major number of publications in the present top 100 belongs to the USA, as well as previous bibliometric reviews in the field of dentistry ^18^. The country accounted for almost 30% of the number of citations, highlighting the important financial resources to research and many of the research centers ^(^ ^7^ ^,^ ^9^. Brazil was the second country with the highest number of published papers, with 20% of the total number of citations. Thus, together these countries were responsible for half of the most cited papers on this topic. Furthermore, we highlight the two institutions with the highest number of contributions: Indiana University - located in the USA - and the State University of Ponta Grossa - located in Brazil. North America was the continent with the highest number of publications, which is in line with the most prevalent country. However, the second continent with the highest number of publications was Europe which, even not including the second prominent country, included other countries that together provided this visibility for the European continent.

Among the authors with the highest number of papers, Cochran MA and Matis BA stands out, mainly interventional studies on bleaching techniques and agents. Sulieman M, another prominent author, all studies as the first author, worked mainly on laboratory studies on techniques and bleaching agents and the interaction of these agents with the dental structure. Louguercio AD and Reis A, authors who also obtained a significant number of publications, mainly intervention studies on tooth whitening side effects such as tooth sensitivity, root resorption and toxicity. Among the authors with the highest number of publications, Cochran MA and Matis BA led the number of citations, both with 569 citations. However, Attin T and Rotstein I, with 5 publications each, obtained a higher number of citations, 624 and 588 respectively. Joiner A, with 3 publications, obtained merit for being the author with the highest number of citations in this top 100, with 653 citations, being the only author of the most-cited paper.

An extensive variety of bleaching agents are available and their adverse effects, such as tooth wear, were investigated in the literature, mainly in laboratory-based studies ^19^. This may explain why laboratory-based studies were the most prevalent in the top 100, followed by intervention studies and literature reviews. Systematic reviews of randomized controlled trials represent the highest level of scientific evidence ^10^. However, only three studies were systematic reviews and only one was based exclusively on randomized clinical trials, the other two also included laboratory studies and review. The systematic review and meta-analysis performed by He et al. ^(^ ^22^, evaluated the influence of light on bleaching efficacy and tooth sensitivity during in-office vital bleaching and showed that after nearly 30 years of tooth bleaching, there were only 11 randomized clinical trials of sufficient quality to be included in the meta-analysis. In this paper, the authors highlight that more studies are needed to help clinicians understand the effects and risks associated with bleaching agents and procedures. This finding corroborates with this review, where only three systematic reviews were identified, and only one performed a meta-analysis of data. For Epple et al. ^(^ ^20^, the major challenge in planning and conducting clinical studies investigating tooth bleaching is related to the sample pattern and selection, since tooth color is generally related to the patient's habits and may vary within the sample.

The risk of harmful effects of bleaching agents on tooth structure has received attention in the last years ^(^ ^3^. Some of the included studies have shown that aggressive treatments can alter the surface integrity and microstructure of enamel crystals ^(^ ^19^ ^,^ ^20^. Thus, the interaction of bleaching agents with dental tissues was the most prevalent topic in this bibliometric analysis. Another topic frequently discussed in the most-cited papers was bleaching techniques and agents. There is a variety of bleaching products available, mainly based on hydrogen peroxide and carbamide peroxide ^(^ ^19^. These agents are supplied in different concentrations and are used in different techniques, which have different treatment times and durations ^23^. In addition, one of the main complaints of patients undergoing tooth bleaching is tooth sensitivity ^(^ ^23^ ^,^ ^24^. Thus, another addressed topic was the adverse effects of tooth bleaching. Tooth sensitivity and oral irritation are the most common side effects, despite being mild and transient ^(^ ^24^. An important discussion related to tooth bleaching is the use of light during treatment. A systematic review and meta-analysis performed to assess the influence of light on tooth bleaching effectiveness and tooth sensitivity showed that light increased the risk of sensitivity and did not improve the whitening effect ^(^ ^22^.

The strengths of this top 100 are related to the pioneering of addressing tooth bleaching, as no previous bibliometric reviews have been conducted on the subject. The assessment of the most studied topic, the main authors who collaborated on the scientific development, and the most frequent study designs may assist researchers to identify gaps in the literature on tooth bleaching that need to be explored in future studies. As a limitation, it can mention that the use of WoS-CC database alone may not have contemplated all high-cited papers on the topic. However, the decision to use the WoS-CC was based on other bibliometric analyzes in dentistry ^(^ ^8^ ^,^ ^9^ as it is one of the main recommended bases for this type of study ^(^ ^25^. 

In conclusion, the top 100 most-cited papers related to tooth bleaching were mainly published by the USA and Brazil, with laboratory-based studies addressing topics related to the effects of bleaching agents on tooth structure being the most prevalent. This bibliometric review reveals the main characteristics of the most-cited papers that studied tooth bleaching over the years, as well as recognizes the authors and institutions that contributed to the scientific development in this field of knowledge.

## References

[1] Rodríguez-Martínez J, Valiente M, Sánchez-Martín MJ (2019). Tooth whitening: from the established treatments to novel approaches to prevent side effects. J Esthet Restor Dent.

[2] Silva FBD, Chisini LA, Demarco FF, Horta BL, Correa MB (2018). Desire for tooth bleaching and treatment performed in Brazilian adults: findings from a birth cohort. Braz Oral Res.

[3] Epple M, Meyer F, Enax J (2019). A Critical Review of Modern Concepts for Teeth Whitening. Dent J.

[4] Joiner A (2016). The bleaching of teeth: a review of the literature. J Dent.

[5] Fasanaro TS (1992). Bleaching teeth: history, chemicals, and methods used for common tooth discolorations. J Esthet Dent.

[6] Moodley J, Singh V, Kagina BM, Abdullahi L, Hussey GD (2015). A bibliometric analysis of cancer research in South Africa: study protocol. BMJ Open.

[7] Baldiotti ALP, Amaral-Freitas G, Barcelos JF, Freire-Maia J, Perazzo MF, Freire-Maia FB, Paiva SM, Ferreira FM, Martins-Júnior PA (2021). The Top 100 Most-Cited Papers in Cariology: A Bibliometric Analysis. Caries Res.

[8] Rocha AO, Santos PS, Machado BA, Bolan M, Cardoso M, Martins-Júnior PA, Santana CM (2022). The Top 100 Most-Cited Papers in Erosive Tooth Wear: A Bibliometric Analysis. Caries Res.

[9] Paschoal MAP, Belém FV, Clementino LC, Martins-Júnior PA (2022). Application of lasers in dentistry: a bibliometric study of the top 100 most-cited papers. Braz Oral Res.

[10] Buchalla W, Attin T (2007). External bleaching therapy with activation by heat, light or laser--a systematic review. Dent Mater.

[11] Rotstein I, Dankner E, Goldman A, Heling I, Stabholz A, Zalkind M. (1996). Histochemical analysis of dental hard tissues following bleaching. J Endod.

[12] Wang Y, Wen X, Jia Y, Huang M, Wang F, Zhang X, Bai Y, Yuan G, Wang Y (2020). Piezo-catalysis for nondestructive tooth whitening. Nat Commun.

[13] Kwon SR, Wertz PW (2015). Review of the Mechanism of Tooth Whitening. J Esthet Restor Dent.

[14] Seale SN, McIntosh JE, Taylor AN (1981). Pulpal reaction to bleaching of teeth in dogs. J Dent Res.

[15] Pritchard A (1969). Statistical Bibliography or Bibliometrics?. J.Doc.

[16] Gonçalves AP, Plá AL, Rodolfo B, Nahsan FP, Correa MB, Moraes RR. (2019). Top-100 Most Cited Dental Articles with Authors from Brazil. Braz Dent J.

[17] Feijoo JF, Limeres J, Fernández-Varela M, Ramos I, Diz P (2014). The 100 most cited articles in dentistry. Clin Oral Investig.

[18] Melo G, Flausino CS, Darella IK, Miguel AFP, Martins PA, Rivero ERC (2022). Top 100 most-cited articles on intraoral squamous cell carcinoma and its risk factors: a bibliometric study. Braz Oral Res.

[19] Eachempati P, Kumbargere Nagraj S, Kumar Kiran, Krishanappa S, Gupta P, Yaylali IE (2018). Home-based chemically induced whitening (bleaching) of teeth in adults. Cochrane Database Syst Rev.

[20] Epple M, Meyer F, Enax J (2019). A Critical Review of Modern Concepts for Teeth Whitening. Dent J.

[21] Joiner A (2010). Whitening toothpastes: a review of the literature. J Dent.

[22] He LB, Shao MY, Tan K, Xu X, Li JY (2012). The effects of light on bleaching and tooth sensitivity during in-office vital bleaching: a systematic review and meta-analysis. J Dent.

[23] Carneiro AMP, Barros APO, de Oliveira RP, de Paula BLF, Silva AM, de Melo Alencar C, Silva CM (2022). The effect of photobiomodulation using low-level laser therapy on tooth sensitivity after dental bleaching: a systematic review. Lasers Med Sci.

[24] Costa JB, McPharlin R, Hilton T, Ferracane JI, Wang M (2012). Comparison of two at‐home whitening products of similar peroxide concentration and different delivery methods. Operative Dentistry.

[25] Ionescu S, Madge OL, Robu I, Brătucu E, Daha C (2021). Surgical Oncology in Romania: An Analysis of Research and Impact Based on Literature Search in PubMed and Web of Science. Biomed Res Int.

